# A model-based prediction of the calcium responses in the striatal synaptic spines depending on the timing of cortical and dopaminergic inputs and post-synaptic spikes

**DOI:** 10.3389/fncom.2013.00119

**Published:** 2013-09-13

**Authors:** Takashi Nakano, Junichiro Yoshimoto, Kenji Doya

**Affiliations:** ^1^Neurobiology Research Unit, Okinawa Institute of Science and Technology Graduate UniversityOkinawa, Japan; ^2^Neural Computation Unit, Okinawa Institute of Science and Technology Graduate UniversityOkinawa, Japan; ^3^Graduate School of Information Science, Nara Institute of Science and TechnologyNara, Japan

**Keywords:** striatal medium spiny neuron, calcium signaling, spike-timing-dependent plasticity, dopamine modulation, multi-compartment model

## Abstract

The dopamine-dependent plasticity of the cortico-striatal synapses is considered as the cellular mechanism crucial for reinforcement learning. The dopaminergic inputs and the calcium responses affect the synaptic plasticity by way of the signaling cascades within the synaptic spines. The calcium concentration within synaptic spines, however, is dependent on multiple factors including the calcium influx through ionotropic glutamate receptors, the intracellular calcium release by activation of metabotropic glutamate receptors, and the opening of calcium channels by EPSPs and back-propagating action potentials. Furthermore, dopamine is known to modulate the efficacies of NMDA receptors, some of the calcium channels, and sodium and potassium channels that affect the back propagation of action potentials. Here we construct an electric compartment model of the striatal medium spiny neuron with a realistic morphology and predict the calcium responses in the synaptic spines with variable timings of the glutamatergic and dopaminergic inputs and the postsynaptic action potentials. The model was validated by reproducing the responses to current inputs and could predict the electric and calcium responses to glutamatergic inputs and back-propagating action potential in the proximal and distal synaptic spines during up- and down-states. We investigated the calcium responses by systematically varying the timings of the glutamatergic and dopaminergic inputs relative to the action potential and found that the calcium response and the subsequent synaptic potentiation is maximal when the dopamine input precedes glutamate input and action potential. The prediction is not consistent with the hypothesis that the dopamine input provides the reward prediction error for reinforcement learning. The finding suggests that there is an unknown learning mechanisms at the network level or an unknown cellular mechanism for calcium dynamics and signaling cascades.

## 1. Introduction

The striatum is the input region of the basal ganglia and receives glutamate input from the cortex and dopaminergic input from the midbrain (Smith and Bolam, 1990). The plasticity of the the cortico-striatal synapses is affected by the timing between the presynaptic dopamine inputs, presynaptic (cortical) glutamatergic inputs and the postsynaptic (striatal) action potentials, which is known as the spike-timing-dependent plasticity (STDP) (Fino et al., 2005; Pawlak and Kerr, 2008; Shen et al., 2008). Among these, the dopamine input is regarded as the reward prediction error and plays a critical role in the reinforcement learning theory in the basal ganglia (Reynolds et al., 2001; Doya, 2002). While the learning theory predicts that the dopamine input following the spike output induces plasticity, the effect of timing of dopamine input on STDP is still unknown. The dopamine-dependent plasticity can be mediated directly by activation of the intracellular signaling cascades within the synaptic spines and indirectly by the modulation of the synaptic receptors and the membrane currents that affect the calcium influx to synaptic spines. We previously proposed a computational model of the intracellular signaling cascades and showed that the direction and the strength of the plasticity are determined by the amplitude and relative timing between the dopamine input and calcium concentration within the synaptic spines (Nakano et al., 2010).

The calcium concentration has intrinsically complex dynamics and is dependent on multiple factors including the calcium influx through ionotropic glutamate receptors, the intracellular calcium release by activation of metabotropic glutamate receptors, and the opening of calcium channels by EPSPs and back propagating action potentials. Furthermore, dopamine is known to modulate the efficacies of NMDA receptors, some types of the calcium channels, and sodium and potassium channels that affect the back propagation of action potentials. Accordingly, the striatal synaptic plasticity is known to be modulated by the spontaneous oscillations of the postsynaptic membrane potential between the up-state and down-state (Charpier and Deniau, 1997) and by the location of the synapse on the dendrite (Kampa et al., 2007). Here we have constructed a morphologically realistic electric compartment model of the striatal medium spiny neuron and we predict the calcium responses in the synaptic spines with variable timings of the glutamatergic and dopaminergic inputs and the postsynaptic action potentials. The model is based upon our own imaging and 3D reconstruction of medium spiny neurons and upon previous models of ionic and synaptic currents, calcium dynamics, and their dopaminergic modulation. We investigated the calcium responses in the proximal and distal synaptic spines during up- and down-states under various timing parameters for glutamate, dopamine, and postsynaptic action potentials using numerical simulations.

This paper is organized as follows: In Section 2, we present the specification of the model constructed for this study. In Section 3, we demonstrate that the model well reproduces electrophysiological properties reported in the literature, to support the validity of the model. Subsequently, we predict the intracellular calcium responses to various timing of the triplet inputs: presynaptic glutamate and dopamine inputs, and a postsynaptic spike. Herein, we show the distribution of the calcium sources to clarify which factors predominantly contribute to calcium responses. Finally, we summarize the main findings of this study and discuss their relationship with the corticostriatal synaptic plasticity in Section 4.

## 2. Methods

### 2.1. Morphological modeling

**Figure 1:**
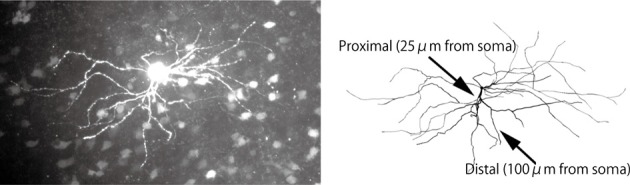
**Morphology of a medium spiny neuron expressing D1-type dopamine receptors. Top**: A medium spiny neuron filled with the fluorescent dye Alexa 488 and observed using a DSU confocal microscope. **Bottom**: Morphological data imported into the NEURON simulator. The arrow heads indicate the proximal and distal dendrites described in Section 2.1.

An electric compartment model was constructed using realistic morphology based on measurements from actual medium spiny neurons. This allowed us to precisely evaluate the effects of back-propagating action potentials. To obtain morphological images, acute corticostriatal slices (300 μm thickness) were prepared from p21-25 Drd1a eGFP Swiss Webster mice (Gong et al., 2003). A neuron was filled with biocytin through a patch pipette and tagged with Alexa 488. A 3D morphological image (Figure 1) was obtained using the Neurolucida neuronal tracing system with a DSU confocal microscope. Cell morphology was manually traced using Neurolucida. The traced data included information regarding the lengths and diameters of the dendrites (Hines and Carnevale, 2001), and were converted to NEURON hoc files using NLMorphologyViewer and NLMorphologyConverter software^1^. In this process, all spines were ignored.

To measure calcium transients in different spines, two spines of diameter 1 μm and length 1.273 μm (i.e., volume of 1 μm^3^) were attached to the proximal and distal dendrites (see arrows in Figure 1), which were located on 25 μm and 100 μm away from the soma, respectively.

### 2.2. Multi-compartment model

The electric properties of the neuron was approximated by splitting the dendrites into small compartments. The membrane potential of the *j*th compartment, denoted by *V*_*j*_, was represented as:

(1) $$\begin{array}{l} C_{j}\frac{dV_{j}}{dt}=-I_{\text{leak},\;j}-I_{\text{chan},\;j}-I_{\text{syn},\;j}-I_{\text{stim},\;j}\\ \;+g_{j-1,j}\left(V_{j-1}-V_{j}\right)-g_{j,j+1}\left(V_{j}-V_{j+1}\right). \end{array}$$

Here, *I*_leak, *j*_ = *g*_leak, *j*_(*V_j_* − *E*_leak_) is a leak current through passive channels of the *j*th compartment, *g*_leak, *j*_ is passive transmembrane conductance, and *E*_leak_ is the passive reversal potential. *I*_chan, *j*_ and *I*_syn, *j*_ are ionic currents through active channels and synaptic channels in the *j*th compartment, respectively (See the details in Section 2.3). *I*_stim, *j*_ is a current induced by external stimuli (e.g., current injection in whole-cell patch clamp experiments in Section 3.1). The last two terms in Equation (1) are membrane currents from neighboring compartments connected by coupling conductance *g*_*j* − 1, *j*_ and *g*_*j, j* + 1_, respectively. The coupling conductance *g*_*j, j* + 1_ is calculated as 1/*g*_*j, j* + 1_ = 2*l*_*j*_/(*g_a_*π *d*^2^_*j*_)+ 2*l*_*j* + 1_/(*g*_*a*_π *d*^2^_*j* + 1_), where *d*_*j*_ and *l*_*j*_ are the diameter and length of *j* th compartment. Spatial discretization of the model neurons was completed automatically according to the d_lambda rule implemented in the NEURON simulator (Hines and Carnevale, 2001). The neuron is decided to 156 compartments.

The effects of the spines ignored in the morphological modeling were mimicked by locally adjusting the conductance per area *g*_leak, *j*_ and the capacitance per area *C*_*j*_ based on the area ratio of the membrane surfaces between the dendrites and the spines (Holmes, 1989; Koch, 1998). More specifically, we let *F*_*j*_ be defined as *F*_*j*_ = 1 + *A*_spine_, *j/ A*_dend, *j*_, where *A*_dend, *j*_ and *A*_spine, *j*_ are the membrane surface area of the dendrites and the spines in the *j*th compartment, respectively. We set *g*_leak, *j*_ = *g*_leak_ *F_j_* and *C*_*j*_ = *CF_j_*, where *g*_leak_ and *C* is the passive transmembrane conductance and the membrance capacitance averaged over the whole neuron, respectively^2^. The parameters used for the multi-compartment model are shown in Table 1.

**Table 1:** **Parameters of compartmodel**.

***C***	**Membrane capacitance**	**1 μ*F* cm^−2^**
*g*_leak_	Passive transmembrane conductance	1.7e-5 S cm^−2^
*E*_leak_	Leak reversal potential	−70 mV
*g_a_*	Axial conductance	0.01 Scm
*F_j_*	Area ratio of dendrites over spines	
	if *j* is a proximal component	1
	if *j* is a middle component	1.3
	if *j* is a distal component	3

These values were based on Wolf et al. (2005).

### 2.3. Ionic and synaptic currents

The ionic and synaptic currents were modeled based on (Wolf et al., 2005; Moyer et al., 2007). In this section, we briefly review the original model and clarify our modifications.

The model included the following ionic channels: fast (NaF) and persistent (NaP) sodium channels; inwardly rectifying (KIR), slow A-type (KAs), fast A-type (KAf), 4-AP resistant persistent (KRP), small conductance calcium-dependent (SK) and large-conductance calcium-dependent (BK) potassium channels; N-(CaN), Q- (CaQ), R- (CaR), L-type Cav 1.2 (Cav1.2), L-type Cav 1.3 (Cav1.3), and T- (CaT) calcium channels (Catterall, 2000). For each compartment, the total current through the ionic channels was given by

(2a) $$I_{\text{chan}}=I_{\text{NaChan}}+I_{\text{KChan}}+I_{\text{CaChan}}$$

(2b) $$I_{\text{NaChan}}=\mu_{\text{NaF}}I_{\text{NaF}}+\mu_{\text{NaP}}I_{\text{NaP}}$$

(2c) $$\begin{array}{l} I_{\text{KChan}}=\mu_{\text{KIR}}I_{\text{KIR}}+\mu_{\text{KAs}}I_{\text{KAs}}+\mu_{\text{KAf}}I_{\text{KAf}}\\ \;+\mu_{\text{KRP}}I_{\text{KRP}}+\mu_{\text{SK}}I_{\text{SK}}+\mu_{\text{BK}}I_{\text{BK}} \end{array}$$

(2d) $$\begin{array}{l} I_{\text{CaChan}}=\mu_{\text{CaN}}I_{\text{CaN}}+\mu_{\text{CaQ}}I_{\text{CaQ}}+\mu_{\text{CaR}}I_{\text{CaR}}\\ \;+\mu_{\text{Cat}}I_{\text{CaT}}+\mu_{\text{Cav1}.\text{2}}I_{\text{Cav1}.\text{2}}+\mu_{\text{Cav1}.\text{3}}I_{\text{Cav1}.\text{3}}, \end{array}$$

where the index of the compartment, *j*, is omitted for notational simplicity ^3^. *I*_NaChan_, *I*_KChan_, and *I*_CaChan_ are the currents summed over sodium, potassium, and calcium channels, respectively. The coefficient μ_*z*_ for each channel-type *z* is the dopamine modulation factor, and the details are described in Section 2.5. For the moment, we assume that μ_*z*_ = 1 is fixed for each channel-type *z* so that the dopamine modulation can be ignored for simplicity.

The current through each type *z* of sodium and potassium channel was given by

(3) $$I_{z}={\bar{g}}_{z}x_{z}(t,V)\left(V-E_{\text{rev}}\right),$$

where ḡ_*z*_ is the maximum conductance of the channel, and *E*_rev_ is the reversal potential that was set to *E*_rev_ = 50 mV for every sodium channel and *E*_rev_ = −90 mV for every potassium channel. *x*_*z*_(*t, V*) is the time- and voltage-dependent variable that summarizes the activation and inactivation states of the channel *z* given by the Hodgkin-Huxley formulation [See the detail in Supplementary Material of (Moyer et al., 2007)].

The current through each type *z* of calcium channel, was given by the Goldman-Hodgkin-Katz (GHK) current formulation:

(4) $$I_{z}=P_{z}\frac{4F^{2}V}{RT}\frac{{\left[{\text{Ca}}^{2+}\right]}_{\text{i}}-{\left[{\text{Ca}}^{2+}\right]}_{\text{o}}\exp\{-2FV/(RT)\}}{1-\exp\{-2FV/(RT)\}}$$

where the gas constant, Faraday constant, and the temperature were set to *R* = 8.31 J/mol/K, *F* = 96489 C/mol, and *T* = 303.15 K (equivalently, 30°C), respectively. [Ca^2+^]_i_ and [Ca^2+^]_o_ are the concentrations of intracellular and extracellular calciums, respectively. [Ca^2+^]_o_ was constant at 5 m M, while [Ca^2+^]_i_ varied over time *t* (Section 2.4). *P*_*z*_ is the calcium permeability of the channel *z*, given by

(5) $$P_{z}={\bar{p}}_{z}x_{z}(t,V),$$

where ${\bar{p}}_{z}$ is the maximum permeability and *x*_*z*_(*t, V*) has the same meaning as Equation (3).

The model also included AMPA-type glutamate receptors (AMPARs) and NMDA-type glutamate receptors (NMDARs). For each compartment, the total postsynaptic current through the receptors, denoted by *I*_syn_, was given by

(6) $$I_{\text{syn}}=\mu_{\text{AMPA}}I_{\text{AMPA}}+\mu_{\text{NMDA}}I_{\text{NMDA}},$$

where the current though each receptor *z*, *I*_*z*_, was given by the same form as Equation (3), and the reversal potential was set to *E*_rev_ = 0 mV for both receptors.

In our study, the variable *x*_*z*_(*t, V*) for every *z* had the same dynamics as (Moyer et al., 2007). The only difference was the setting of the maximum conductance ḡ_*z*_ and the maximum permeability ${\bar{p}}_{z}$, and we adjusted these parameters to fit our experimental data using Neurofitter (Geit et al., 2007). The resulting parameters are listed in Table 2.

**Table 2:** **Parameters for ionic and synaptic currents**.

**Channel-type *z***		***ḡ_z_*(S cm^−2^)**	**Channel-type *z***	**${\bar{p}}_{z}$ (cm/s)**
NaF	(s)	1.96	Cav1.2	1.34e-5
	(p,m,d)	0.0185	Cav1.3	1.7e-6
NaP	(s)	7.36e-5	CaN	2.0e-5
	(p,m,d)	2.86e-7	CaQ	1.2e-5
KAf	(s,p)	0.247	CaR	5.2e-5
	(m,d)	0.0429	CaT	8.0e-7
KAs	(s,p)	0.0129		
	(m,d)	9.44e-4	**Receptor-type *z***	***ḡ_z_*(pS)**
KIR		4.18e-4	AMPA	447
KRP		7.3e-3	NMDA	226
BK		1.58e-3		
SK		0.0910		

The parameters for NaF, NaP, KAf and KAs channels varied depending on the compartment location. Thus, the characters s, p, m and p attached to the channel-type stand for the soma, and proximal, middle, and distal dendrites, respectively.

### 2.4. Calcium dynamics

**Figure 2:**
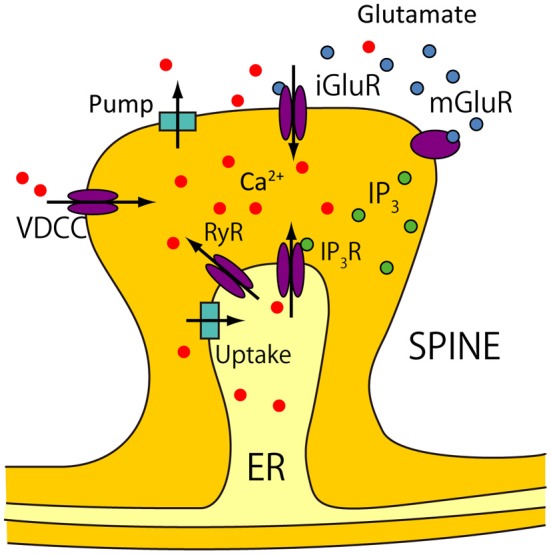
**Calcium dynamics**. Calcium sources are divided into two groups. One group consists of extracellular sources, and includes calcium influx from voltage-dependent calcium channels (VDCCs), and from calcium permeable ionotropic glutamate receptors (iGluRs). The iGluRs in our model consists of AMPARs and NMDARs. Calcium is also pumped out of cells into the extracellular matrix by calcium pumps. The other group consists of intracellular calcium stores in the endoplasmic reticulum (ER), which are accessed by IP_3_ receptors (IP_3_Rs) and ryanodine receptors (RyRs). IP_3_ is produced by metabotropic glutamate receptors (mGluRs). Calcium uptake is mediated by the Sarco/ER Ca^2+^-ATPase (SERCA), and leaks occur through the ER.

The model proposed in Wolf et al. (2005); Moyer et al. (2007) does not consider calcium release from intracellular calcium stores [i.e., endoplasmic reticulum (ER)] through ryanodine and inositol-1,4,5-triphosphate (IP_3_) channels, which is suggested to significantly affect the activity of various neurons (Falcke et al., 2000; Varona et al., 2001b,a). To precisely evaluate the calcium responses in two spines attached to the proximal and distal dendrites, we refined the calcium dynamics model based on De Schutter and Smolen (1998). Figure 2 shows all the processes contributing to calcium dynamics in our model.

More specifically, the transient change in intracellular calcium [Ca^2+^]_i_ were given by

(7) $$\begin{array}{l} \frac{d{\left[{\text{Ca}}^{2+}\right]}_{\text{i}}}{dt}=J_{\text{CICR}}+J_{{\text{IP}}_{3}}-J_{\text{uptake}}+J_{\text{leak}}+J_{\text{cur}}-J_{\text{pump}}\\ \;+\left({\left[{\text{Ca}}^{2+}\right]}_{\infty }-{\left[{\text{Ca}}^{2+}\right]}_{\text{i}}\right)/\tau . \end{array}$$

Here, *J*_CICR_ is the flux caused by calcium-induced calcium release (CICR) from intracellular stores. This process is mediated by ryanodine receptors, and was modeled as

(8) $$J_{\text{CICR}}=V_{\text{CICR}}\frac{{\left[{\text{Ca}}^{2+}\right]}_{\text{i}}}{{\left[{\text{Ca}}^{2+}\right]}_{\text{i}}+K_{\text{CICR}}}\left({\left[{\text{Ca}}^{2+}\right]}_{\text{ER}}-{\left[{\text{Ca}}^{2+}\right]}_{\text{i}}\right)\text{​},$$

where *V*_CICR_ = 3 × 10^−12^ s^−1^ is the maximum rate of CICR and *K*_CICR_ = 0.2 μM. [Ca^2+^]_ER_ = 0.20 mM is the calcium concentration in the ER.

*J*_IP3_ is the flux caused by IP_3_-induced calcium release from intracellular stores. It is known that the process has a bell-shaped steady state curve that depends on [Ca^2+^]_i_ with a sharp peak around 0.2 μM; thus, it was modeled as

(9) $$J_{{\text{IP}}_{3}}=V_{{\text{IP}}_{3}}m^{3}h^{3}\left({\left[{\text{Ca}}^{2+}\right]}_{\text{ER}}-{\left[{\text{Ca}}^{2+}\right]}_{\text{i}}\right)\text{​},$$

where *V*_IP3_ = 1 × 10^−9^ s^−1^ is the maximum rate of the IP_3_-induced release. *m* and *h* are an activation gate and an inactivation gate, respectively. They were given by

(10) $$m=\frac{{\left[{\text{IP}}_{3}\right]}_{\text{i}}}{{\left[{\text{IP}}_{3}\right]}_{\text{i}}+d_{{\text{IP}}_{3}}}\frac{{\left[{\text{Ca}}^{2+}\right]}_{\text{i}}}{{\left[{\text{Ca}}^{2+}\right]}_{\text{i}}+d_{\text{act}}},$$

(11) $$h=\frac{d_{\text{inh}}\left({\left[{\text{IP}}_{3}\right]}_{\text{i}}+d_{{\text{IP}}_{3}}\right)}{d_{\text{inh}}\left({\left[{\text{IP}}_{3}\right]}_{\text{i}}+d_{{\text{IP}}_{3}}\right)+{\left[{\text{Ca}}^{2+}\right]}_{\text{i}}\left({\left[{\text{IP}}_{3}\right]}_{\text{i}}+d_{\text{dis}}\right)},$$

where *d*_IP3_ = 0.13μM, *d*_act_ = 8.2 × 10^−2^ μM, *d*_inh_ = 1.05μM, and *d*_dis_ = 0.94 μM are the dissociation constants for IP_3_ binding to the uninhibited receptors, Ca^2+^-activation of the receptors, Ca^2+^-inhibition of the receptors, and disinhibition of the Ca^2+^-inhibited receptors, respectively. [IP_3_]_i_ is the level of intracellular IP_3_. It should be noted that IP_3_ is generated via G-proteins when glutamate binds to metabotropic glutamate receptors. The transient change in [IP_3_]_i_ was modeled by

$$\frac{d{\left[{\text{IP}}_{3}\right]}_{\text{i}}}{dt}=\gamma_{{\text{IP}}_{3}}{\hat{t}}_{\text{Glu}}\exp\left(-{\hat{t}}_{\text{Glu}}/\tau_{{\text{IP}}_{3}}\right)-\beta_{{\text{IP}}_{3}}\left({\left[{\text{IP}}_{3}\right]}_{\text{i}}-{\left[{\text{IP}}_{3}\right]}_{\text{min}}\right)\text{​},$$

where ${\hat{t}}_{\text{Glu}}$ is the time elapsed since the last glutamate release from the presynaptic neuron, and τ_IP__3_ = 220 ms is the time to peak. γ_IP__3_ = 5× 10^−6^ mM/ms^2^ determines the maximum rate of IP_3_ production, β_IP__3_ = 0.2 ms^−1^ is the removal rate, and [IP_3_]_min_ = 0.24 μM is the minimum level of [IP_3_]_i_.

*J*_uptake_ is the calcium uptake to the ER, which was modeled as

(12) $$J_{\text{uptake}}=V_{\text{uptake}}\frac{{\left[{\text{Ca}}^{2+}\right]}_{\text{i}}^{2}}{K_{\text{uptake}}^{2}+{\left[{\text{Ca}}^{2+}\right]}_{\text{i}}^{2}},$$

where *V*_uptake_ = 0.75 × 10^−9^ μM/s is the maximum rate of uptake and *K*_uptake_ = 0.2 μM is the dissociation constant.

*J*_leak_ is the calcium leak from the ER, which was modeled as

(13) $$J_{\text{leak}}=V_{\text{leak}}({\left[{\text{Ca}}^{2+}\right]}_{\text{ER}}-{\left[{\text{Ca}}^{2+}\right]}_{\text{i}}),$$

where *V*_leak_ = 6.15 × 10^−14^ s^−1^ is the maximum rate of leak.

*J*_cur_ is the calcium current through calcium channels and calcium permeable glutamate receptors, and was given by

(14) $$J_{\text{cur}}=-\frac{I_{\text{CaChan}}+c_{\text{AMPA}}I_{\text{AMPA}}+c_{\text{NMDA}}I_{\text{NMDA}}}{2Fv},$$

where *F* = 96489 C/mol is Faraday's constant, *v* = 1 μm^3^ is the volume of the compartment. *c*_AMPA_ and *c*_NMDA_ are the effectiveness of calcium ions in the synaptic currents through AMPARs and NMDAs, respectively. They were set to *c*_AMPA_ = 0.0005 μ_AMPA_ and *c*_AMPA_ = 0.01 μ_AMPA_.

*J*_pump_ is pumping activity to the outside of the cell,

(15) $$J_{\text{pump}}=V_{\text{pump}}\frac{{\left[{\text{Ca}}^{2+}\right]}_{\text{i}}}{{\left[{\text{Ca}}^{2+}\right]}_{\text{i}}+K_{\text{pump}}},$$

where *V*_pump_ = 0.1 μM/ms is the time constant of the pump and *K*_pump_ = 0.1 μM is the equilibrium calcium value.

The last term in Equation (7) is a simple diffusion or buffering process, in which the parameters were set to the same as (Wolf et al., 2005), that is, τ = 43 ms and [Ca^2+^]_∞_ = 0.01 μM.

### 2.5. Dopamine modulation

**Figure 3:**
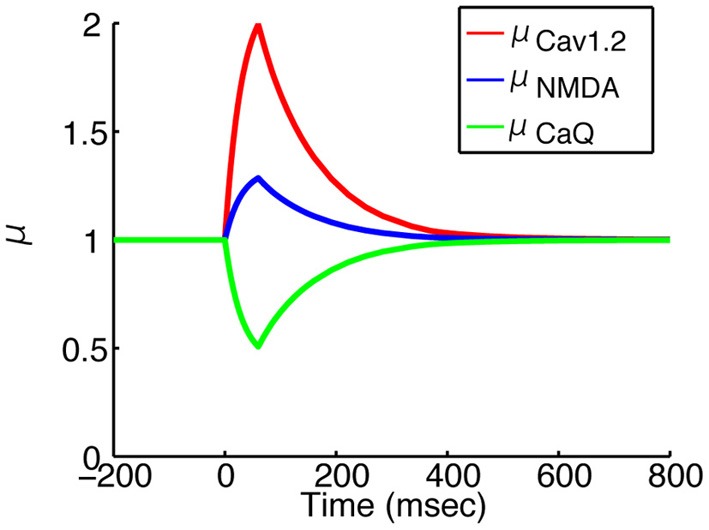
**Time courses of dopamine modulation factors**. Red, blue and green lines indicates μ_Cav1.2_, μ_NMDA_ and μ_CaQ_, respectively. The onset is the time when the dopamine input arrived. All of the factors reache their own peak level in 60 ms, and returns the basal level in 500 ms.

While the effects of dopamine on channel conductance vary in different cell types and brain regions (Surmeier et al., 1995; Yang and Seamans, 1996; Hernández-López et al., 1997; Cepeda et al., 1998; Nicola et al., 2000; Johnson et al., 2003; Surmeier et al., 2007; Steephen, 2011; Zhou and Antic, 2012), the dopamine enhances KIR, Cav1.2 and NMDAR conductances and reduces NaF, CaN and CaQ conductances in the striatum, as summarized in Moyer et al. (2007). To reflect the findings, the dopamine modulation factor μ_*z*_ for each channel/receptor type *z* was variable in our model. Based on Gruber et al. (2003), the transient was modeled as

(16) $$\mu_{z}=\{\begin{array}{ll} \mu_{\text{peak},\;z}+\left(1-\mu_{\text{peak},\;z}\right)\exp\left(-\frac{{\hat{t}}_{\text{DA}}}{\tau_{\text{inc}}}\right) & \text{if }{\hat{t}}_{\text{DA}}<t_{\text{peak}}\\ 1+\left(\mu_{\text{peak},\;z}-1\right)\exp\left(-\frac{{\hat{t}}_{\text{DA}}-t_{\text{peak}}}{\tau_{\text{dec}}}\right) & \text{if }{\hat{t}}_{\text{DA}}\geq t_{\text{peak}}, \end{array}$$

where ${\hat{t}}_{\text{DA}}$ is the time elapsed since the last dopamine input arrived. μ_peak, *z*_ is the peak level of μ_*z*_ and *t*_peak_ is the time to reach the peak. τ_inc_ and τ_dec_ are the time constants in the increasing and decreasing phases of μ_*z*_, respectively. The peak level μ_peak, *z*_ for each channel/receptor type is listed in Table 3. The other parameters were set to *t*_peak_ = 60 ms, τ_inc_ = 30 ms, and τ_dec_ = 100 ms. Figure 3 shows the typical behaviors of μ_*z*_ for some types of channels or receptors.

**Table 3:** **Parameters for dopamine modulation factors**.

**Channel *z***	**μ_peak, *z*_**
NaF	0.95
KIR	1.25
Cav1.2	2.0
CaN	0.2
CaQ	0.5
receptor *z*	μ_peak, *z*_
NMDA	1.3

These values were based on Moyer et al. (2007).

In addition to channel conductance, dopamine affects the voltage dependence of the activation gating of Cav1.3 ionic channels (Moyer et al., 2007). We modeled it in the same manner as the conductance modulation ^4^.

## 3. Results

### 3.1. Voltage and calcium responses: model validation

**Figure 4:**
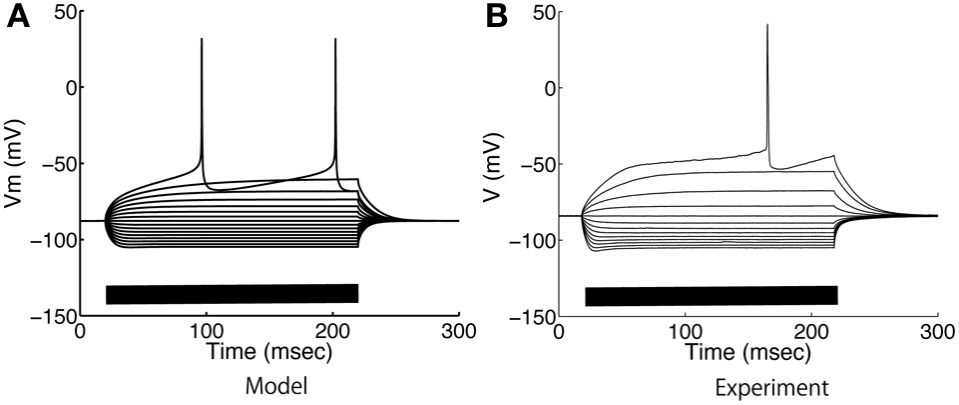
**Somatic voltage responses to step current applications. (A)** Model and **(B)** experimental responses of medium spiny neurons to step current applications from −0.3 nA to 0.28 nA at intervals of −0.04 nA. The bars on the horizontal axes indicate the duration of current applications.

**Figure 5:**
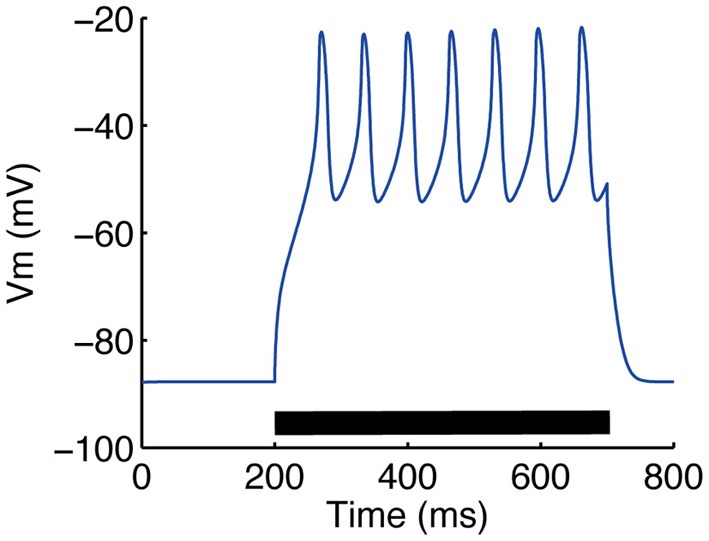
**Somatic calcium transient reproduced by our model**. Calcium spikes following a 0.3 nA step current were observed. To mimic the application of 4-AP (a potassium channel blocker) and TTX (O'Donnell and Grace, 1993), the conductances of KAf, KAs, KRP and NaF channels were multiplied by 0.6, 0.4, 0.5 and 0.25, respectively.

The model parameters were calibrated to fit the electrophysiological properties of medium spiny neurons expressing D1-type dopamine receptors, which were examined by whole-cell patch clamp experiments *in vitro*. Figure 4 compares the membrane potential responses of the model to those of a real neuron. The model replicated the characteristic properties of the medium spiny neurons, with a resting membrane potential around −85 mV, small voltage responses to hyperpolarizing currents, and shallow after-hyperpolarization (AHP) following spike firing. In addition, the model reproduced calcium spikes observed in the experimental condition during a step current input with the application of 4-AP (potassium channel blocker) and TTX (O'Donnell and Grace, 1993) (Figure 5).

The voltage and calcium responses to a back-propagating postsynaptic action potential (bAP) or a single glutamate input were investigated in the spines on proximal and distal dendrites of the model neuron. The membrane potential of medium spiny neurons with intact cortical input fluctuates between the “down-state” of approximately −85 mV and the “up-state” of approximately −65 mV (Wilson and Kawaguchi, 1996). The up-state was simulated by a steady current input of 0.2 nA to the soma and the down-state was simulated by no current input.

**Figure 6:**
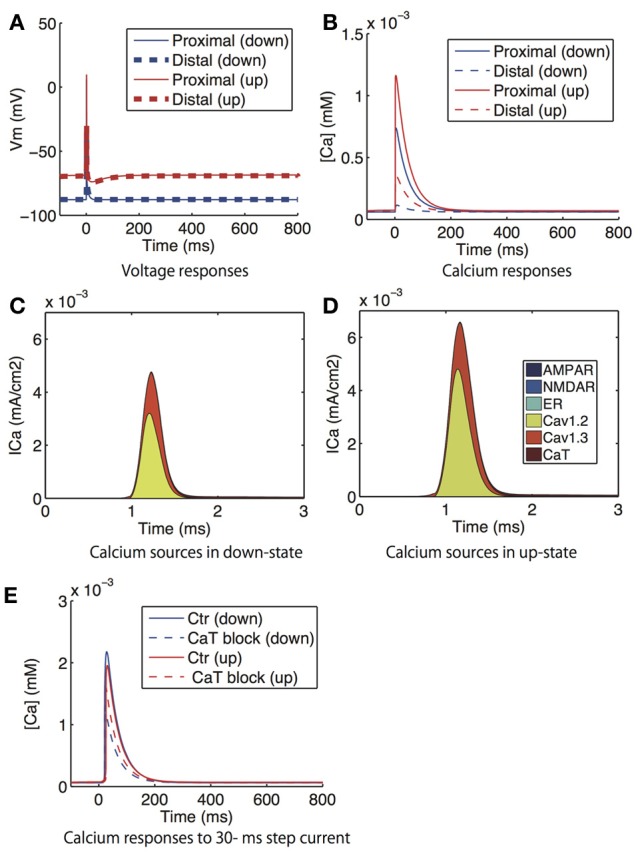
**Model prediction of voltage and calcium responses to bAP in dendritic spines. (A)** Voltage and **(B)** calcium responses in the proximal (solid lines) and distal (dashed lines) dendritic spines to supra threshold 2 ms step currents to the soma, which corresponds to the experiment that investigated the attenuation of bAP-evoked calcium with distance from the soma (Day et al., 2008). The blue lines indicate the down-state and the red lines indicate the up-state simulated by the step current. **(C,D)** shows the distribution of the sources of calcium influx to the proximal spine in the down- and up-states. **(E)** Calcium responses in the proximal dendric spines to a 30 ms step current to the soma in the absence (solid lines) and presence (dashed lines) of T-type calcium channel blockade. Again, the red and blue lines indicates the down-state and the up-state, respectively.

Figure 6 shows the voltage and calcium responses in the dendritic spines to bAPs, where each postsynaptic action potential was evoked by a 2 ms step current application to the soma. The bAPs arrived in the spine without delay, but their amplitudes were attenuated with distance from the soma (Figure 6A). Corresponding to the attenuation, the amplitudes of the calcium responses were also attenuated with distance from the soma (Figure 6B). Calcium responses in the up-state were bigger than in the down-state because of greater calcium influx through L-type calcium (Cav1.2 and Cav1.3) channels in the up-state (Figures 6C,D). However, interestingly, when a wide step current (30 ms duration) was applied to evoke an action potential, the calcium response was smaller in the up-state than in the down-state (Figure 6E), which is consistent with the experimental result (Carter and Sabatini, 2004). In contrast, when T-type calcium channels were blocked, the calcium transients evoked by the wide current pulse were larger in the up-state than in the down-state. Although calcium currents predominantly moved through T-type calcium channels at the moment of the current input, T-type calcium channels in the up-state were inactivated so rapidly that this inversion phenomenon was engendered.

**Figure 7:**
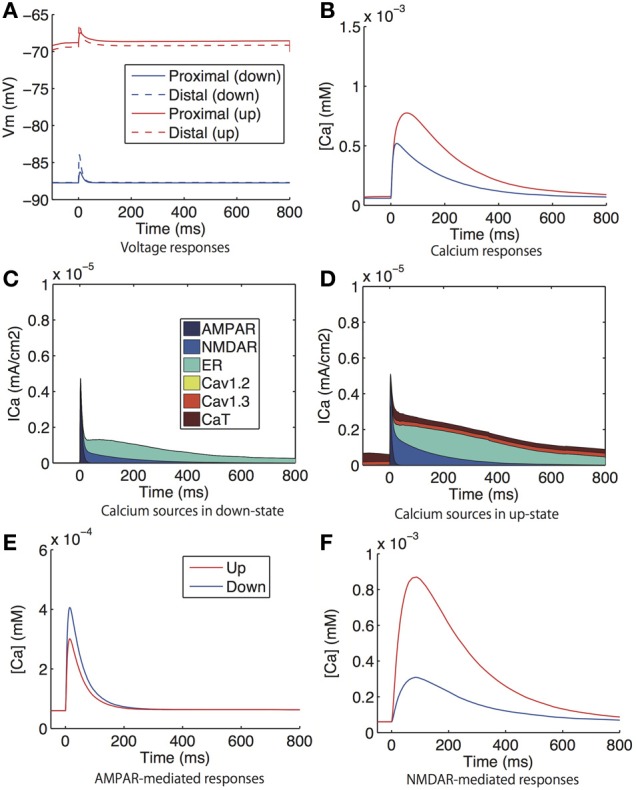
**Model prediction of voltage and calcium responses to glutamate input in spines. (A)** Voltage and **(B)** calcium responses in proximal (solid lines) and distal (dashed lines) dendritic spines to glutamate input. The corresponding experiment was reported in Carter and Sabatini (2004), where the the glutamate input was evoked by uncaged glutamate. **(C,D)** shows the distribution of the sources of calcium influx to the proximal spine **(C)** in the down-state and **(D)** in the up-state. **(E)** AMPAR-mediated calcium signals under the conditions of the sodium, calcium, and NMDAR currents being set at zero. **(F)** NMDAR-mediated calcium responses under the conditions of the sodium, calcium, and AMPAR currents being set at zero. Glutamatergic synaptic inputs were applied to the same spines in which calcium was observed. In **(A,B,E,F)**, the blue lines indicate the down-state and the red lines indicate the up-state simulated by a steady current input.

Figure 7 shows the voltage responses (i.e., excitatory postsynaptic potentials; EPSPs) and calcium responses in the dendritic spines to a single glutamate input. Here, the inputs were applied to the same spines in which the responses were measured. There were no significant difference in EPSPs or calcium responses between the proximal and distal spines, and the EPSPs and calcium responses were greater in the up-state (Figures 7A,B). The increased calcium influx in the up-state was mediated by voltage-gated calcium channels (CaT and Cav1.3 channels), NMDARs, and calcium-dependent calcium release from the ER (Figures 7C,D).

In (Carter and Sabatini, 2004), the following experimental findings were reported: (1) The AMPAR-mediated calcium response was larger in the down-state than in the up-state during the application of CPP (NMDAR antagonist), TTX (sodium channel blocker), and VDCC blockers; and (2) the NMDAR-mediated calcium response was larger in the up-state than in the down-state, and it was smaller in the up-state than in the down-state in magnesium-free conditions in the presence of NBQX (AMPAR antagonist), TTX, and VDCC blockers. We simulated the experiments in the following manners: For the Experiment 1), we fixed the sodium, calcium, and AMPAR currents in our model at zero and measured the calcium responses to glutamate input. Similarly, for Experiment 2), we fixed the sodium, calcium, and NMDAR currents at zero. Figures 7E,F show the simulation results, which accurately reproduced the properties reported in (Carter and Sabatini, 2004).

When calcium responses to glutamate input and to bAP are compared, glutamate input made a larger response than bAP in the proximal spine and smaller responses in the distal spine. This amplitude relationship was also observed in the experimental study Carter and Sabatini (2004), even though a conflicting finding, which glutamate input made a smaller response than bAP, has also been reported recently (Shindou et al., 2011).

### 3.2. Timing-dependent calcium responses to paired inputs

Using the model, we predicted how the calcium responses depend on the relative timing of paired inputs: a presynaptic input (either glutamate (Glu) or dopamine (DA)) and a postsynaptic spike (Post). Hereafter, the time difference between Glu and Post is denoted by Δ*t*_Glu_ where Δ*t*_Glu_ > 0 if Glu precedes Post. For more intuitive notations, we also use “Glu-Post” and “Post-Glu” for Δ*t*_Glu_ > 0 and Δ*t*_Glu_ < 0, respectively. The notational rules are applied to the time difference between DA and Post, where the above-mentioned “Glu” are all replaced by “DA.”

**Figure 8:**
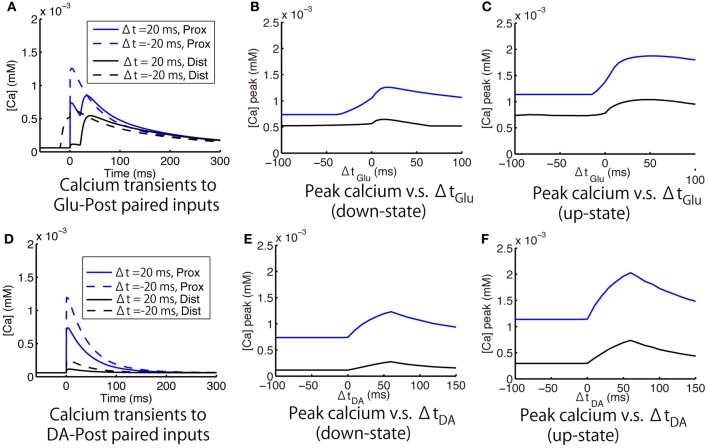
**Model prediction of timing-dependent calcium responses to paired input. (A–C)**. Calcium responses that depended on the timing of glutamate synaptic input (Glu) relative to a postsynaptic spike (Post). **(A)** Typical calcium responses to Glu preceding (solid lines) and following (dashed lines) Post by 20 ms. **(B,C)** Peak of the calcium transient as a function of Δ*t*_Glu_ (time difference between Glu and Post) in the down- and up-states, respectively. **(D–F)** Calcium responses that depended on the timing of dopamine synaptic input (DA) relative to Post. **(D)** Typical calcium responses to DA preceding (solid lines) and following (dashed lines) Post by 20 ms. **(E,F)** Peak of the calcium transient as a function of Δ*t*_Glu_ (time difference between DA and Post) in the down- and up-states, respectively. For all panels, blue and black lines indicate the measurements at the proximal and distal dendritic spines, respectively. Post was evoked by a 2 ms postsynaptic step current.

Figure 8A shows the calcium responses to Glu preceding or following Post by 20 ms in the down-state. Figures 8B,C shows timing-dependent peak calcium responses as a function of Δ*t*_Glu_ in the down- and up-states, respectively. In both the up- and down-states, Glu-Post engendered higher calcium responses than Post-Glu. As for the depencence on the location of spines, the amplitude of calcium responses was larger in the proximal dendrite than in the distal dendrite, but a tendency toward timing dependence was preserved in both spines. The effects of DA timing relative to Post on calcium responses were similar to those of Glu timing: Namely, DA-Post engendered a larger calcium response than Post-DA (Figures 8D–F).

**Figure 9:**
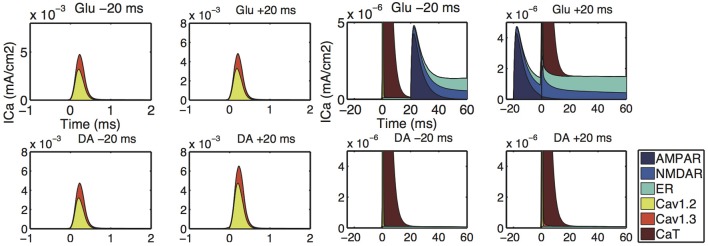
**Input-timing-dependent calcium sources predicted by our model. Upper** and **lower panels** show the distribution of the sources of calcium influx corresponding to Figures 8A,D, respectively. The left 2-by-2 panels are the simulation results for Δ*t*_Glu_ = −20 **(upper-left)**, Δ*t*_Glu_ = + 20 **(upper-right)**, Δ*t*_DA_ = −20 **(lower-left)**, Δ*t*_DA_ = + 20 **(lower-right)**, respectively. The magnifications are shown in the right 2-by-2 panels.

The simulation shows sources of calcium influx (Figure 9). The increased calcium response to a Glu-Post condition was caused by enhanced calcium influx through NMDARs (Figure 9 upper panels). This is consistent with the calcium imaging experiment investigating the calcium response to the time difference between the glutamate input and bAP (Shindou et al., 2011). The simulation results showed that the calcium release from ER helps to sustain the long-lasting calcium increase by glutamate, and emphasizes the difference in the calcium responses between Glu-Post and Post-Glu conditions. The increased calcium response to DA-Post was caused by enhanced calcium influx through L-type calcium (Cav1.2 and Cav1.3) channels (Figure 9 lower panels).

### 3.3. Triplet interaction

**Figure 10:**
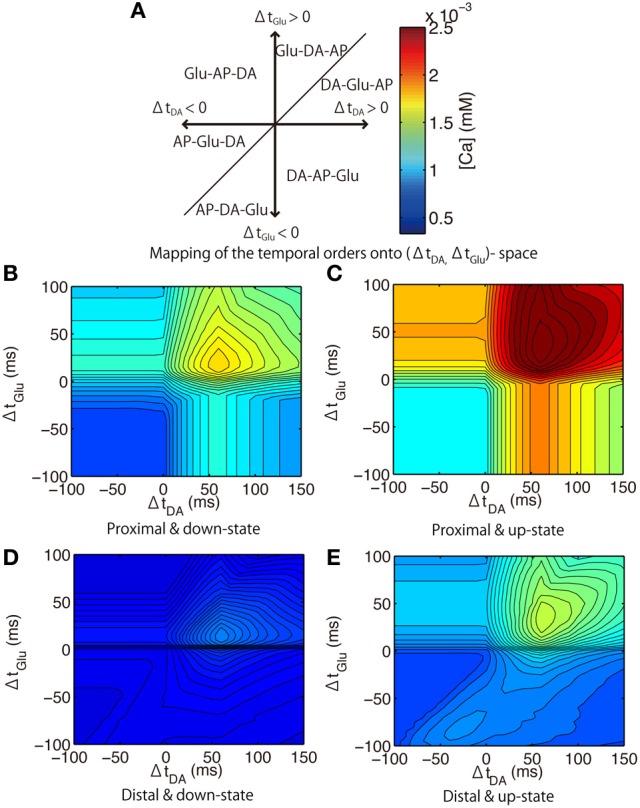
**Model prediction of calcium responses to triplet-timed inputs. (A)** illustrates the relationship between each value of (Δ*t*_DA_, *t*_Glu_) and the temporal order of triplet inputs: DA; Glu; and Post. For example, the timing area 1 (upper-right) indicates that the the temporal order of triplet inputs was DA, Glu, Post from the first. The color bar indicates the peak of calcium response, which is common for the **(B–E)** and Figure 3. **(B–E)**. Peak calcium concentrations in the proximal spine **(B,C)** or distal spine **(D,E)** during the down-state **(B,D)** or the up-state **(C,E)**.

We then investigated the dependence of calcium responses on the timing of triplet inputs: Glu, DA and Post. Figure 10A shows the mapping of the temporal order of the triplet inputs onto the (Δ*t*_DA_, Δ*t*_Glu_)-space. Figures 10B–E shows the peak calcium concentration in the proximal and distal spines as a function of (Δ*t*_DA_, Δ*t*_Glu_). In the both down- and up-states, the effects of dopamine inputs were the most prominent when DA preceded Post by approximately 60 ms, and amplified more when Glu preceded Post. While this tendency was also observed in the both proximal and distal spines, the peak amplitude was lower in the distal spines than in the proximal spines.

**Figure 11:**
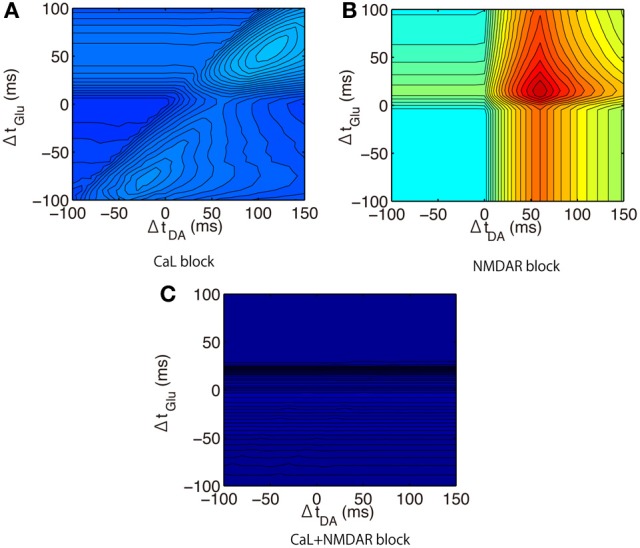
**Model prediction of calcium responses to triplet-timed inputs under blockade conditions. (A)** L-type calcium channels (CaL) blockade condition where both conductances of Cav1.2 and Cav1.3 were set to zero. **(B)** NMDAR blockade condition where conductance of NMDAR was set to 0 pS. **(C)** Combinations of blockade conditions **(A)** and **(B)**.

We then predicted the calcium responses in the proximal spines when different synaptic channels were blocked. When L-type calcium (Cav1.2 and Cav1.3) channels were blocked, the peak amplitude approached that in the distal spines (Figure 11A). This suggests that the weaker amplitudes in the distal spines were caused by insufficiently large bAPs to activate the L-type calcium channels (Figure 6). The timing dependence of Glu and Post was weakened by blocking NMDARs (Figure 11B). The timing dependence of DA and Post was eliminated when both NMDARs and L-type calcium channels were blocked (Figure 11C). It is noteworthy that the results showed that calcium responses to the triplet inputs cannot be explained by just a linear sum of DA- and Glu-derived effects. Rather, calcium levels were drastically elevated only when both Glu and DA adequately preceded Post. The optimal timing to induce the largest calcium response was (Δ*t*_Glu_, Δ*t*_DA_) = (+50 ms, +60 ms) in the up-state, but it shifted to (Δ*t*_Glu_, Δ*t*_DA_) = (+20 ms, +60 ms) in the down-state because of the non-linear interaction between DA- and Glu-derived effects.

### 3.4. Dopamine-mediated modulation of NMDARs

An uncaging calcium study in the cerebellum reported that the amplitude of long-term depression was well predicted from a leaky integration of the intracellular calcium concentration (Tanaka et al., 2007). The leaky integration *x* of calcium concentration is defined as

(17) $$\tau \frac{dx}{dt}=-x+a{\left[{\text{Ca}}^{2+}\right]}_{\text{i}}$$

where τ is the time constant of integration, and *a* is a scaling factor that converts the calcium concentration into the transduction signal regulating the synaptic efficacy. We set to τ = 600 ms and *a* = 20 and evaluated the peak amplitude of *x* as a function of (Δ*t*_Glu_, Δ*t*_DA_).

**Figure 12:**
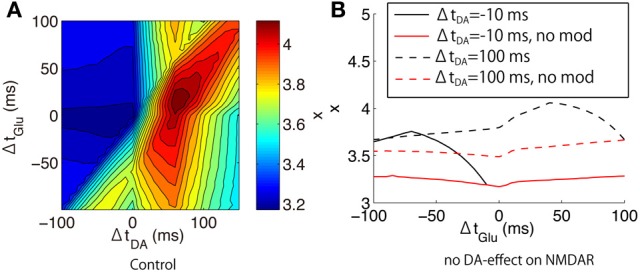
**Leaky integration of calcium responses to triplet-timed inputs predicted by our model. (A)** The peak amplitude of calcium leaky integrator *x* as a function of (Δ*t*_Glu_, Δ*t*_DA_), which were evaluated in the proximal spines in the up-state. **(B)** The difference between the original model (black lines) and the modified model where all DA effects on NMDAR were excluded (red lines). The solid and dashed lines indicate the different DA-timings: Δ*t*_DA_ = −10 ms and Δ*t*_DA_ = +100 ms, respectively.

Interestingly, the dependence of the leaky integration *x* on the glutamate and dopamine input timings was different from that of the peak calcium concentration (Figure 12A). The timing between dopamine and glutamate is more important than the timing between glutamate and bAP. The DA timing modulated the leaky integrator response to Glu-Post bidirectionally: Glu-Post enhanced *x* in the DA-Post condition but Post-Glu enhanced *x* in the Post-DA condition.

To investigate the mechanisms of the DA-timing modulation, we performed additional simulations where all DA effects on NMDAR were blocked (Figure 12B). In this blockade condition, Glu-Post did not enhance *x* even in the DA-Post timing at Δ*t*_DA_ = +100 ms. Also, the Glu-timing dependence on the leaky integrator almost vanished regardless of the DA-timing.

## 4. Conclusion and discussion

We constructed a multi-compartment model of a medium spiny neuron of the striatum based on real morphological data. The model could reproduce the major electrophysiological properties the neuron and allowed us to predict the calcium responses to timed presynaptic inputs (glutamate and dopamine) and a postsynaptic spike under various conditions, including the up- and down-states. We measured calcium dynamics in both proximal and distal spines and evaluated the peak and leaky integration of calcium.

Our major findings are summarized as follows:

1. Glutamate input preceding a postsynaptic spike induced higher calcium responses than glutamate input following a postsynaptic spike, which was mediated by NMDARs, L-type calcium channels (i.e., Cav1.2 and Cav1.3) and the intracellular calcium store (Figures 8, 9).
2. Dopamine input preceding a postsynaptic spike also induced higher calcium responses than dopamine input following a postsynaptic spike, which was mediated by L-type calcium channels (Figures 8, 9).
3. Although their amplitudes differed, there was no difference in the timing dependence on calcium responses between the up- and down-states. At distal spines, the back-propagating action potential was attenuated, so that the dopamine timing effect was small. Nevertheless, the timing dependence on calcium responses between distal and proximal spines is preserved.
4. The timing dependence of the leaky integration of the calcium was different from that of the peak calcium concentration. The timing of dopamine input could modulate the timing dependency of the glutamate input and postsynaptic spike, which was mediated by dopaminergic modulation of NMDARs (Figure 12).

Calcium regulation in synaptic spines plays a key role in the synaptic plasticity (Artola and Singer, 1993). In the cortico-striatal synapses, dopamine also plays a critical role in inducing long-term potentiation by increasing cAMP and activating PKA in the cells expressing D1 receptors (Nakano et al., 2010). Our model shows that dopamine regulates intracellular calcium changes, and suggests the possibility that dopamine regulates synaptic plasticity through calcium in parallel with the PKA-related cascade. In particular, dopamine preceding a postsynaptic spike could increase the leaky integration of calcium induced by glutamate and a postsynaptic spike, and make it easier to potentiate the synaptic efficacy. In addition, our results show that application of dopamine before glutamate induces the largest calcium responses.

From the perspective of reinforcement learning theory, dopamine is hypothesized to be the reward prediction error signal that reinforces the association between the sensory information encoded in the glutamatergic input from the cortex and the action realized by the striatal spike output. Based on this hypothesis, the reinforcement signal should follow the sensory input and action output, but our results are inconsistent with this prediction. An alternative hypothesis suggests that dopamine codes salience rather than reward prediction error (Redgrave and Gurney, 2006; Berridge, 2007): The dopamine is released even by unexpected sensory events that have no obvious appetitive reinforcement consequence (Redgrave and Gurney, 2006), or by novel stimulations that trigger a motivational state, i.e., “wanting” for the reward (Berridge, 2007). According to this scenario, our simulation results might suggest that the dopamine signal amplifies the striatal response (i.e., attention) to the coincident events (including action selection) coded by presynaptic glutamate input, and marks the input as biologically significant events. However, the effective temporal order of the dopamine-glutamate input in our simulation is still inconsistent with this alternative theory, as well as with the reinforcement learning theory. Irrespective of which hypothesis is correct, this inconsistency suggests that there is an unknown network mechanism to overcome these timing constraints, or else there is a missing link in the dopaminergic signaling cascades. A multilevel study integrating calcium dynamics and intracellular signal transduction may be critical to elucidating these mechanisms.

Our contribution to the model is the refinement of the previous studies (Wolf et al., 2005; Moyer et al., 2007) by incorporating (1) real morphological data; (2) a kinetic model of calcium release from intracellular calcium stores; and (3) the time variation of the dopamine modulation. It turned out that the difference between the presence and the absence of a kinetic model of calcium release was significant in the timing effect of glutamate and bAP (Figure 9). On the other hand, in this study, real morphological data do not makes a significant difference in calcium dynamics between these two models with the sophisticated and simplified morphology, while morphological variability leads to variability of neural activity in other neurons (Mainen and Sejnowski, 1996). The reason would be involved in our assumption that the membrane characteristic of compartments is homogeneous within each of three dendritic regions (proximal, middle and distal). Such validation is out of scope in this study but will be an important issue in the future.

Another important issue to discuss is that there is the other type of the medium spiny neurons in the striatum expressing D2-type dopamine receptors, and showing different projection and morphology (Gertler et al., 2008). Since it is unclear whether the morphological difference is involved in the difference of neural activity, our model will serve as a basic model for D1-type medium spiny neurons to solve this question. Also, our model excludes a direct mechanism of calcium buffers, which is known to affect the electrical properties of neurons and other excitable cells as has been reported previously Torres et al. (2004); Harks et al. (2003). Another important question is whether such a mechanism affects the neural activity in the striatum, though none of the electrophysiological experiments compared with our simulation results employed the calcium buffer.

### Conflict of interest statement

The authors declare that the research was conducted in the absence of any commercial or financial relationships that could be construed as a potential conflict of interest.

## Footnotes

^1^http://www.neuronland.org

^2^In other words, we assumed that the spines had the same channel density as the dendrites.

^3^The same omission will be made later, as long as the dependence is obvious from the context.

^4^Specifically, the parameter *V*_half_ for the activation gate variable *m* of Cav1.3 channels in Moyer et al. (2007) was varied so that *V*_half_ = −33 mV in the resting state and *V*_half_ = −43 mV at the peak. The time course followed the same curve as Figure 3.
